# The relationship between bone density and the oral function in older adults: a cross-sectional observational study

**DOI:** 10.1186/s12877-021-02547-6

**Published:** 2021-10-22

**Authors:** Yoko Hasegawa, Shotaro Tsuji, Koutatsu Nagai, Ayumi Sakuramoto-Sadakane, Joji Tamaoka, Masayuki Oshitani, Takahiro Ono, Takashi Sawada, Ken Shinmura, Hiromitsu Kishimoto

**Affiliations:** 1 Department of Dentistry and Oral Surgery, Hyogo College of Medicine, 1-1 Mukogawa-cho, Nishinomiya, Hyogo Japan; 2 Division of Comprehensive Prosthodontics, Niigata University Graduate School of Medical and Dental Sciences, 5274, Gakkocho-dori 2-bancho, Chuo-ku, Niigata, 951-8514 Japan; 3 Department of Orthopaedic Surgery, Hyogo College of Medicine, 1-1 Mukogawa-cho, Nishinomiya, Hyogo Japan; 4 Department of Physical Therapy, School of Rehabilitation, Hyogo University of Health Sciences, 1-3-6 Minatojima, Chuo-ku, Kobe, Hyogo Japan; 5 Hyogo Dental Association, 5-7-18 Yamamoto-dori, Chuo-ku, Kobe, Hyogo Japan; 6 Division of General Medicine, Department of Internal Medicine, Hyogo College of Medicine, 1-1 Mukogawa-cho, Nishinomiya, Hyogo Japan

**Keywords:** Falling, Oral function, Skeletal muscle mass, Bone density, Physical performance, Older adults

## Abstract

**Background:**

Falls among older adults with a low bone density can lead to a bedridden state. Declining bone density increases the risk of falls resulting fractures in older adults. A person’s physical performance is known to be closely related to bone density, and a relationship between the physical performance and the oral function is also known to exist. However, there currently is a lack of evidence regarding the relationship between bone density and the oral function. We assessed the relationship between the bone density and the both the oral function and physical performance among older adults.

**Patients and methods:**

754 older adults aged 65 years or older who independently lived in rural regions and who were not taking any medications for osteoporosis participated. We checked all participants for osteoporosis using an ultrasonic bone density measuring device. Regarding the oral function, we evaluated the following factors: remaining teeth, occlusal support, masticatory performance, occlusal force, and tongue pressure. We also evaluated body mass index (BMI) and skeletal muscle mass Index as clinical characteristics. The normal walking speed, knee extension force and one-leg standing test were evaluated as physical performance. For the statistical analyses, we used the Mann–Whitney U test, chi-square test, the Kruskal-Wallis, and a multiple regression analysis.

**Results:**

Eighty-one percent of the females and 58% of the males had osteoporosis or a decreased bone mass. The occlusal force, masticatory performance and the tongue pressure showed significant association with the bone density. The participants physical performance showed a significant association with their bone states except for walking speed. According to a multiple regression analysis, clinical characteristics (sex, age, BMI), one-leg standing and occlusal force showed independent associations with the bone density. It was suggested that the bone density tends to increase if the occlusal force is high and/or the one-leg standing test results are good.

**Conclusions:**

The bone density in the older adults showed a significant relationship not only with clinical characteristics or physical performance, but also with occlusal force. It may also be effective to confirm a good oral function in order to maintain healthy living for older adults.

## Introduction

Falling is an extremely serious event for older adults, requiring high medical expenses for treatment. It is common knowledge that a decline in the muscle strength of older adults can lead to a decreased activity level in daily living [1–3], such as a deterioration of balance and a decreased walking ability [2, 4], which can lead to a shorter life expectancy, while also preventing older adults from living fulfilling lives due to a decreased quality of life (QOL) [3].

Moreover, it is common knowledge that the bone density decreases with age, and that, in females in particular, the decrease in the production of estrogen following menopause can result in a sudden decrease in bone density [5]. Falls that occur when an individual has a decreased bone density can easily lead to bone fractures, thus shortening the life expectancy of older adults [2, 6]. One in three individuals aged 65 and older experience at least one fall per year. It has been reported that 10 to 15% of these falls lead to a major injury [4, 7]. In the United States, falling is a major cause of emergency hospital visits for individuals age 65 and older, with 70% of fatal accidents among this age group related to falling [8]. In Japan, the number one medical problem requiring nursing care is musculoskeletal disorders (24.6%) such as fractures and articular diseases caused by falling. In particular, fall fractures among older adults with osteoporosis account for 12.1% [9]. Further, it has been reported that older adults who have fallen once have a high risk of falling again [6]. Therefore, preventing falling is important socially as well as in terms of medical costs [10].

It has been thought that the cause of falling in older adults is a decreased physical performance; however, a decreased physical performance is known to be related to a decreased oral function [11–13]. Oral frailty is a risk factor for developing physical frailty [11, 13]. It has been demonstrated that the risk of physical frailty is associated with a reduced occlusal force [14, 15], and a healthy dental condition at 75 year of age will have the risk of becoming frail in later life [16]. We have been involved in a cohort study on older adults individuals who live independently in rural area in Japan. Based on our results, we have been reporting the relationship between the risk of falling and physical frailty with oral function [11, 12, 17–19]. We conducted further studies on the risks of falling in older adults and the oral function and have suggested that preventing tooth loss and maintaining occlusal force can decrease the risk of falling [19].

Our survey has been evaluated the physical performance, such as walking speed and balance of the trunk of the body. We also measured the bone density, and measured the body composition of all subjects as clinical characteristics. We have reported that oral functions are closely related to physical performance and clinical characteristics of older adults [11, 17, 19] . Our previous report also showed that occlusal force, which is an index of oral function, was positively correlated with knee extension force and/or walking speed [19]. The relationship between bone density and physical characteristics such as body weight has already been recognized [20]. It has been reported that bone tissue is constantly changing and is a dynamic tissue that adapts and responds to stimuli, including exercise [3, 21]. Namely, although the bone density changes dynamically under the influence of various factors [20, 22], there is a significant relationship between bone density and the physical performance. Therefore, we believe that a relationship may exist between bone density and the oral function. On the other hand, investigations into the relationship between oral function and bone density are insufficient, with many points remaining unclear.

Therefore, this study aimed to identify the relationship between the bone density which is related to the occurrence of fractures after falling and the oral function/physical performance of older adults.

## Material and methods

This cross-sectional study was approved by the institutional review board of Hyogo College of Medicine (approval no. hi-0342) and is part of the Frail Elderly in the Sasayama-Tamba Area (FESTA) study [17–19]. This study was conducted in accordance with the principles of the Declaration of Helsinki. The purpose of the FESTA study is to clarify associations between lifestyle habits and frailty in older adults. The Hyogo College of Medicine Sasayama Medical Center is a branch hospital of the Hyogo College of Medicine, located in the Tanba-Sasayama area of Hyogo, Japan. Therefore, this study was conducted in the Sasayama area.

### Participants

This cross-sectional study used data collected in 2015 to 2018 for the purposes of a cohort study of a medical/dental academic survey. The subjects consisted of 853 older adults aged 65 or older, who participated in the survey targeting older adults who live independently (who have not been using nursing insurance or who only receive mild nursing care) in Tanba-Sasayama City, Hyogo Prefecture, study from June 2015 to December 2018. As the criteria for excluding subjects, we excluded persons who taking medications for treatment of osteoporosis, did not consent to having their oral function evaluated and persons who could not have their body composition measured due to the existence of a pacemaker, individuals with missing data for any assessment items or who had a decreased cognitive function (Mini-Mental State Examination (MMSE) score < 20) [23]. The final number of subjects was 754 participants (male: 276; female: 478; age: 73.4 ± 5.9 years, mean ± SD). We explained the objective and methods of the study to subjects and received their consent in writing in advance. Sasayama City is a city with a population of 42,696 (as of the end of September 2016) and is located in a mountainous region of Hyogo Prefecture. The primary industry of this region is agriculture. It has a noticeably aged population, with older adults aged 65 and older making up 31.4% of the population.

We recruited participants in this academic study through inserts in the local newspaper and through advertising posters at the Hyogo College of Medicine Sasayama Medical Center, with participants voluntarily applying to participate.

### Evaluation of bone density and body composition

For bone density, we used an ultrasonic bone density measuring device (CM-200, FURUNO ELECTRIC) to measure the bone density of the right fibular tarsal bone. We then obtained the value upon comparing the ultrasonic propagation velocity (speed of sound; hereinafter, “SOS”, represented as m/s) in the fibular tarsal bone with the SOS of younger individuals (Young Adult Mean; hereinafter, “YAM”, represented as a percentage). Moreover, based on the criteria for diagnosing osteoporosis, we classified cases with a YAM value of less than or equal to 80 as decreased bone mass, less than or equal to 70 as osteoporosis, and all others as normal [24].

We evaluated the skeletal muscle using a body composition analysis device (Inbody 770, InBody Japan) to perform an impedance analysis using the bio-electrical impedance analysis method and calculated the Skeletal Muscle Mass Index (hereinafter “SMI”) [25] as the index of the skeletal muscle mass of the four limbs. We also calculated the body mass index (hereinafter, “BMI”) using the heights and weights that we measured. The BMI, SMI, age, and sex were defined to the “clinical characteristics”.

### Evaluation of the physical performance

We conducted a study concerning the physical performance related to falling.

During the walking speed test, we told participants to walk at their normal speed. We recorded the normal walking speed (m/sec) (hereinafter, “walking speed”) and analyzed it. We also considered increases or decreases in the walking speed from the start of walking to the end of walking and classified the walking range as a total of 12 m of the measurement range (10 m), from 1 m from the front of the measurement range to 1 m from the end [26].

To measure the knee extension force, we used a manual muscle strength meter (mobie, SAKAI Medical). The subjects were asked to sit upright during the measurements, with their knee joints bent at a 90-degree angle, in order to prevent their gluteal region from rising up from the observation chair. We measured their dominant leg twice, then evaluated the maximum torque value (N) of the two measurements [27].

For the retention time of standing on one leg with eyes open (hereinafter, “one-leg standing”), we started measuring from the instant the dominant leg of the subject left the floor and both of their hands touched their waist. We measured the time (in seconds) until either the patient’s hands moved away from their waist, the position of their foot changed, or a part of their body aside from their supporting foot touched the floor [28]. For the one-leg standing test, we set the maximum time to 60 s.

### Evaluation of the oral function

The subjects sat in reclinable nursing chairs and received oral examinations under sufficient artificial lighting. We evaluated data concerning the number of remaining teeth, occlusal force, occlusal support, masticatory performance, and tongue pressure.

The number of remaining teeth (hereinafter, “remaining teeth”) is the number of teeth, including residual teeth and wisdom teeth.

For the occlusal force, we measured the maximum occlusal force of the first left and right molars using an occlusal force meter (Occlusal Force-Meter GM10, NAGANO KEIKI) and evaluated the sum of the two [29]. The measuring condition in the event the first molar is missing was that the measurement position could be retained at the top and bottom of the tooth while measuring at the position closest to the first molar. For subjects who use dentures, we took the measurements while they were wearing dentures. We also used the following formula to find the balance in occlusal force between the left and right sides (hereinafter, “occlusal balance”).

$$\mathrm{Occlusal}\ \mathrm{balance}\ \left(\%\right)=\left(|\mathrm{difference}\ \mathrm{between}\ \mathrm{left}\ \mathrm{and}\ \mathrm{right}\ \mathrm{occlusal}\ \mathrm{force}|/\mathrm{sum}\ \mathrm{of}\ \mathrm{left}\ \mathrm{and}\ \mathrm{right}\ \mathrm{occlusal}\ \mathrm{force}\right)\times 100.$$

For the masticatory performance, we used gummy jelly for measuring the masticatory performance. After the subjects freely chewed it 30 times, they were asked to spit it out [30]. For tongue pressure, we measured the maximum tongue pressure twice using a JMS Tongue Pressure Measuring Device and took the highest value [31]. To evaluate the occlusal support, we classified the groups into three groups using the Eichner classification [32], in accordance with the status of the remaining teeth: Group A had occlusions in four molar regions; Group B had occlusions in one to three molar regions; and Group C had occlusions in no molar regions. We used these three groups for our analyses.

### Statistical analysis

We conducted our analyses on all subjects as a whole and on male and female subjects separately, and set the significance level for each to 5%. For comparisons between two groups, we performed our evaluations using Mann-Whitney’s U-test or χ^2^ test. Differences between the groups were assessed using the Kruskal-Wallis test, and group differences were evaluated with Bonferroni-corrected Mann-Whitney U tests.

We performed a multiple regression analysis to investigate the relationship with bone density (SOS). The dependent variable was SOS, the explanatory variable was significantly related to the bone density by the Kruskal-Wallis test and χ^2^ test, and the adjusted variable was age and sex (Stepwise selection, input 0.05, removal 0.10). A statistical analysis was performed using the IBM SPSS 25.0 software program (IBM Corporation, Armonk, NY, USA).

## Results

### Summary of subjects

There was a large percentage of female subjects among the total of 754 subjects. Moreover, the BMI, SMI, bone density status (SOS and YAM), knee extension force, and occlusal force were significantly higher in males than in females (Table 1). The results indicated that the SOS and YAM values were both significantly lower in females than in males, with 81% of females having osteoporosis or decreased bone mass. On the other hand, 58% of the male subjects had either osteoporosis or a decreased bone mass (Table 1).

**Table 1:** Summary of subjects

Measurement variables	Overall (***n*** = 754)	Male (***n*** = 276)	Female (***n*** = 478)	***P***-value
Clinical characteristics				
Age (years)	72 (68, 77)	73 (69, 77)	72 (68, 77)	0.08
BMI (kg/m^2^)*	22.7 (20.8, 24.4)	23 (21.3, 24.9)	22.2 (20.5, 24.3)	< 0.001
SMI (kg/m^2^)*	6.4 (5.8, 7.2)	7.4 (6.9, 7.8)	6 (5.6, 6.4)	< 0.001
Bone density statue				
SOS (m/s) *	1488 (1474, 1504)	1496 (1483, 1515.3)	1484 (1470.8, 1497.3)	< 0.001
YAM (%)*	74 (68, 82)	77 (71, 87.8)	72 (66, 78)	< 0.001
Osteoporosis	260 (34.5)	65 (23.6)	195 (40.8)	< 0.001
Decreased bone mass	287(38.1)	95 (34.4)	192 (40.2)	
Normal	207 (27.5)	116 (42)	91 (19)	
Physical performance				
Walking speed (m/sec)	1.5 (1.3, 1.6)	1.4 (1.3, 1.6)	1.5 (1.3, 1.6)	0.57
Knee extension force (N)*	339.2(277, 427.4)	457 (362, 523.5)	301 (248.8, 357.9)	*P* < 0.001
One-leg standing (sec)	30 (11, 60)	31.6 (10.7, 60)	29.3 (11, 60)	0.31
Oral function examinations				
Remaining teeth (0–32)	23 (15, 27)	23 (13, 27)	23 (16, 27)	0.78
Occlusal force (kgf)*	50.8 (26.1, 83.8)	58.3 (29.7, 95.9)	47.8 (24.3, 77.8)	0.002
Occlusal balance (%)	19.5 (8.4, 38)	20.4 (8.5, 38.1)	18.6 (8.3, 37.9)	0.25
Masticatory function (score)	5 (2, 6)	5 (2, 6)	4 (2, 6)	0.17
Tongue pressure (kPa)	33.9 (28.2, 38.8)	34 (28.8, 39.7)	33.8 (27.6, 38.6)	0.11
Occlusal support				
Group A	364 (42.7)	116 (41.3)	248 (43.4)	0.8
Group B	338 (39.6)	106 (37.7)	232 (40.6)	
Group C	151 (17.7)	59 (21)	92 (16.1)	

Data show Median (first quartile, third quartile) or the number of people (%)

*BMI:* body mass index, SMI: Skeletal Muscle Mass Index

*SOS:* Ultrasonic propagation velocity in the fibular tarsal bone. YAM: Value compared with the SOS of younger individuals

Occlusal support: Eichner classification. Group A have contact in four occlusal contact regions, Group B have in three to one occlusal contact regions or anterior region only, and Group C have no occlusal contact regions at all

*P*-value: Comparison between males and females. Mann-Whitney’ s U-test or χ^2^ test.*: There is a significant difference between males and females

### Relationship between bone density and the oral function

Table 2 shows the bone density status of these three groups. There was a significant difference in the bone density of the groups, and SOS was lower in females than in males.

**Table 2:** State of bone density

All (754)		Osteoporosis (*n* = 260)	Decreased bone mass (*n* = 287)	Normal (*n* = 207)	P-value
	SOS (m/s)	1469 (1460, 1475.3)	1490 (1485, 1496)	1516 (1509, 1526)	< 0.001
	YAM (%)	65 (61, 68)	75 (73, 77)	88 (84, 94)	< 0.001
Male (276)		Osteoporosis (*n* = 65)	Decreased bone mass (*n* = 95)	Normal (*n* = 116)	P-value
	SOS (m/s)	1473 (1465.5, 1478)	1492 (1487, 1495.8)	1518 (1509, 1529.5)	< 0.001
	YAM (%)	66 (62, 68)	75 (73, 77)	89 (84, 95.8)	< 0.001
Female (478)		Osteoporosis (*n* = 195)	Decreased bone mass (*n* = 192)	Normal (*n* = 91)	P-value
	SOS (m/s)	1468 (1460, 1474)	1489 (1484.5, 1496)	1513 (1509.3, 1522)	< 0.001
	YAM (%)	65 (61, 68)	75 (72.3, 77)	87 (85, 91)	< 0.001

Median (first quartile, third quartile) or the number of people (%)

Based on the diagnostic criteria for osteoporosis, we classified cases with a YAM value of ≤80 as decreased bone mass, ≤70 as osteoporosis, and all others as normal

*P*-value: Kruskal-Wallis test

The results of relationship between bone density and the oral function for all participates, Occlusal force, masticatory performance and the tongue pressure showed significant association with the state of bone (Table 3). In male participates, Occlusal force and masticatory performance showed significant association with the state of bone. On the other hand, in females, a significant relationship was observed between tongue pressure/occlusal support and the bone state.

**Table 3:** Relationship between bone density and oral function

All (754)	Osteoporosis (*n* = 260)	Decreased bone mass (*n* = 287)	Normal (*n* = 207)	Two-group comparison
Remaining teeth (num)	22 (14, 26)	24 (14, 27)	23 (16, 26)	N.S.
Occlusal force (kgf)*	40 (21.9, 73.4)	53.1 (25, 85.4)	60.3 (33.8, 91.6)	a,b
Occlusal balance (%)	21.8 (9.3, 40.3)	17.4 (7.9, 36.6)	20.1 (8.1, 35.8)	N.S.
Masticatory performance (score) *	4 (1, 6)	5 (2, 6)	5 (3, 6)	b
Tongue pressure (kPa) *	32.8 (26.6, 37.8)	34.3 (28.8, 39.1)	34.6 (30, 39.5)	b
*Occlusal support *				
Group A	97 (30.2)	136(42.4)	88(27.4)	N.S.
Group B	107(35.8)	104(34.8)	88(29.4)	
Group C	56(41.8)	47(35.1)	31(23.1)	
Male (276)	Osteoporosis (n = 65)	Decreased bone mass (n = 95)	Normal (n = 116)	Two-group comparison
Remaining teeth (num)	23 (11, 27)	23 (12, 27)	23 (16.3, 27)	N.S.
Occlusal force (kgf) *	42.5 (22.4, 87.9)	53.1 (26.4, 94.3)	66.1 (41.8, 103.6)	a,b
Occlusal balance (%)	21 (8.8, 39)	19.6 (9.4, 39.7)	21.7 (7, 37.1)	N.S.
Masticatory performance (score) *	4 (2, 6)	5 (1, 6)	5 (4, 6)	a
Tongue pressure (kPa)	33.1 (27.1, 38.6)	33.8 (28.8, 39.9)	34.5 (30.7, 40)	N.S.
*Occlusal support*				
Group A	25(21.9)	38(33.3)	51(44.7)	N.S.
Group B	20(19.2)	38(36.5)	46(44.2)	
Group C	20(34.5)	19(32.8)	19(32.8)	
Female (478)	Osteoporosis (n = 195)	Decreased bone mass (n = 192)	Normal (n = 91)	Two-group comparison
Remaining teeth (num)	22 (15, 26)	24 (17, 27)	23 (16, 26)	N.S.
Occlusal force (kgf)	39.8 (21.8, 72.2)	53.5 (24.9, 84.6)	48 (26.6, 78)	N.S.
Occlusal balance (%)	21.9 (9.9, 40.5)	17.1 (7.5, 36.2)	17.1 (8.7, 34.3)	N.S.
Masticatory performance (score)	4 (1, 5)	5 (2, 6)	5 (2, 6)	N.S.
Tongue pressure (kPa) *	32.6 (26.3, 37.5)	34.4 (28.9, 38.9)	35.3 (30, 39)	b
Occlusal support**				
Group A	72(34.9)	98(47.3)	37(17.9)	c
Group B	87(44.6)	66(33.8)	42(21.5)	
Group C	36(47.4)	28(36.8)	12(16.8)	

median (first quartile, third quartile) or number of people (%)

*: Significant difference in Kruskal-Wallis Test. **: Significant difference by χ^2^-test

Two-group comparison: The Mann-Whitney U test was used to compare two groups, and the *P* value was corrected by the Bonferroni method. a: Statistically significant difference between normal group and decreased bone mass group. b: Statistically significant difference between normal group and osteoporosis group. c: Statistically significant difference between the osteoporosis group and the decreased bone mass group. N.S.: No significant difference in all comparisons

### Relationship between bone density and clinical characteristics or physical performance (Table 4)

For all participants (male and female participants) and male participants, clinical characteristics (age, BMI, SMI) or physical performance (knee extension force, and one-leg standing) showed significant associations with the bone density except for normal walking speed. On the other hand, for females, age and knee extension force showed significant associations with the bone density.

**Table 4:** Relationship between bone density and clinical characteristics or physical performance

All (754)	Osteoporosis (n = 260)	Decreased bone mass (n = 287)	Normal (n = 207)	Two-group comparison
Age (years) *	74 (69.3, 78.8)	72 (68,78)	71 (67,75)	a,b,c
BMI (kg/m^2^) *	21.9 (20.3, 24.1)	22.7 (20.7,24.3)	23.1 (21.4,25.4)	a, b
SMI (kg/m^2^)*	6.07 (5.68,6.71)	6.32 (5.79,7.01)	6.9 (6.06,7.65)	a,b
Walking speed (m/sec)	1.48 (1.28,1.6)	1.45 (1.3,1.63)	1.47 (1.35,1.6)	N.S.
Knee extension force (N) *	313 (253, 384)	329.5 (275.5398.1)	391.6 (315.8499)	a,b
One-leg standing (sec) *	24.1 (8.9, 52)	26 (10.5,60)	40 (18.6,60)	a,b
Male (276)	Osteoporosis (n = 65)	Decreased bone mass (n = 95)	Normal (n = 116)	
Age (years) *	76 (71,82)	72 (68,78)	72 (68.3,75)	b
BMI* (kg/m^2^)*	22.6 (20.1,24.8)	22.7 (20.7,24.3)	23.4 (21.7,25.8)	b
SMI (kg/m^2^)*	7.25 (6.63,7.55)	6.32 (5.79,7.01)	7.57 (7.06,8.11)	a,b
Walking speed (m/sec)	1.46 (1.21,1.61)	1.45 (1.3,1.63)	1.47 (1.35,1.59)	N.S.
Knee extension force (N) *	445.7 (337,512)	329.5 (275.5398.1)	486.4 (388.3544)	a,b
One-leg standing (sec) *	15.6 (7,34.2)	26 (10.5,60)	48.8 (19,60)	b
Female (478)	Osteoporosis (n = 195)	Decreased bone mass (n = 192)	Normal (n = 91)	
Age (years) *	74 (69,78)	72 (67,76.8)	69 (67,75)	b
BMI (kg/m^2^)	21.7 (20.3,24)	22.2 (20.5,24.3)	23 (20.9,24.6)	N.S.
SMI (kg/m^2^)	5.88 (5.56,6.32)	5.97 (5.54,6.4)	5.99 (5.7,6.45)	N.S.
Walking speed (m/sec)	1.48 (1.3,1.59)	1.45 (1.3,1.63)	1.49 (1.36,1.61)	N.S.
Knee extension force (N) *	291 (236.3360.8)	300 (246.3352)	320.6 (272,379)	b
One-leg standing (sec)	28.1 (10.6,56.3)	25 (10.6,60)	34.7 (15.6,60)	N.S.

*: Significant difference in Kruskal-Wallis Test. a: Statistically significant difference between normal group and decreased bone mass group, b: Statistically significant difference between normal group and osteoporosis group, c: Statistically significant difference between the decreased bone mass group and the osteoporosis group. N.S.: No significant differences in all comparisons

Kruskal-Wallis test and multiple comparison (Mann-Whitney’s U-test, with corrections to the *P*-values using the Bonferroni method)

### Factors associated with bone density (Table 5)

According to a multiple regression analysis, for all participants, age, sex, BMI, occlusal force and one-leg standing were chosen as a significant independent variable (determination coefficient R^2^ = 0.185). For males, one-leg standing, BMI and occlusal force were chosen as significant independent variables (determination coefficient R^2^ = 0.155). On the other hand, for women the only significant independent variable was age, and the coefficient of determination of the formula was also low (R^2^ = 0.06).

**Table 5:** Factors associated with bone density

All subjects	B	Standard error	β	t-value	P-value	CI Lower-Upper	
Sex (0:Male, 1:Female)	−13.898	1.848	−0.28	−7.519	< 0.001	−17.527	−10.268
Age (years)	−0.741	0.164	−0.185	−4.505	< 0.001	−1.063	− 0.418
BMI (kg/m^2^)	1.06	0.303	0.128	3.494	0.001	0.464	1.656
One-leg standing (sec)	0.116	0.044	0.108	2.631	0.009	0.029	0.203
Occlusal force (kgf)	0.054	0.023	0.09	2.39	0.017	0.01	0.098
(constant)	1522.885	15.418		98.771	< 0.001	1492.608	1553.163
*SMI (kg/m*^*2*^*)*	*0.009*		*0.016*	*0.219*	*0.827*		
*Tongue pressure (kPa)*	*0.024*		*0.023*	*0.607*	*0.544*		
*Masticatory performance (score)*	*0.024*		*0.029*	*0.595*	*0.552*		
*Knee extension force (N)*	*0.033*		*0.044*	*0.832*	*0.406*		
**Males only**	B	Standard error	β	t-value	P-value	CI Lower-Upper	
One-leg standing (sec)	0.156	0.033	0.277	4.670	< 0.001	0.090	0.222
BMI (kg/m^2^)	0.922	0.264	0.205	3.493	0.001	0.402	1.442
Occlusal force (kgf)	0.039	0.018	0.133	2.237	0.026	0.005	0.074
(constant)	50.183	6.249		8.030	< 0.001	37.874	62.492
*Masticatory performance (score)*	*−0.024*		*−0.031*	*−0.372*	*0.71*		
*SMI (kg/m*^*2*^*)*	*0.037*		*0.052*	*0.581*	*0.562*		
*Knee extension force (N)*	*0.016*		*0.016*	*0.247*	*0.805*		
*Age (years)*	*−0.101*		*−0.105*	*−1.599*	*0.111*		
**Females only**	B	Standard error	β	t-value	P-value	CI Lower-Upper	
Age (years)	−0.522	0.125	− 0.245	−4.165	< 0.001	− 0.768	−0.275
(constant)	117.944	9.272		12.720	< 0.001	99.690	136.198
*Tongue pressure (kPa)*	*−0.021*		*−0.022*	*− 0.349*	*0.727*		
*Knee extension force (N)*	*0.09*		*0.097*	*1.492*	*0.137*		
*Occlusal support (reference Group A)*	*–*		*–*	*–*	*–*		
*Group B*	*0.04*		*0.039*	*0.658*	*0.511*		
*Group C*	*−0.027*		*−0.028*	*−0.445*	*0.657*		

The multiple regression analysis was performed as follows: Dependent variable: SOS, explanatory variables: items significantly associated with bone density in Tables 3 and 4, adjusted variables: age, and sex (only All subjects formula) stepwise selection method (input 0.05, removal 0.10). Italicized explanatory variables are variables that have been removed from the final multiple regression formula

B: Unstandardized coefficient, β: Standardized coefficient, CI: 95% confidence interval for unstandardized coefficient

The coefficient of determination: All subjects; R^2^ = 0.185, Males only; R^2^ = 0.155, Females only; R^2^ = 0.06

## Discussion

In this study, we aimed to assess the relationship between the bone density and both the oral function and physical performance among older adults. A decreased bone density makes individuals more susceptible to developing osteoporosis, and osteoporosis leads to increased fractures in the event of a fall, which could lead to a serious medical condition.

In the present study, participant characteristics (sex, age, BMI) and physical performance (one-leg standing) as well as the oral function (occlusal force) showed independent associations with bone density. Regarding the mechanism of bone density change, it is known that bone mineral density increases with the load placed on the skeletal system [33]. Body weight and exercise are the loads applied to the skeletal bones, and it is known that this load increases as the body weight increases and as muscle strength increases through exercise, resulting in increased bone density [34, 35]. It has been reported that bone tissue is constantly changing and is a dynamic tissue that adapts and responds to stimuli, including exercise [3, 21]. Namely, although the bone density changes dynamically under the influence of various factors [22], there is a correlation between bone density and physical performance. Reduced skeletal muscle mass results in decreased physical performance, such as one-leg standing. The etiology of muscle mass loss is multifactorial, but age-related hormonal decline and inactivity have been implicated in its development [36]. Decreased serum testosterone levels have been associated with decreased muscle mass and strength in older men [37, 38]. Experimental mouse models suggest that the masseter muscle is more testosterone-sensitive than the limb skeletal muscles [39]. It is also possible that age-related hormonal changes, such as growth hormone and insulin-like growth factor-1, in conjunction with testosterone, may be involved in the performance of masticatory muscles in older adults [37, 38]. In this study, the association between bone density and occlusal force was stronger in males than in females, suggesting that hormones may have an effect on the masseter muscle, which is involved in occlusal force. Regarding the relationship between physical performance and the oral function, several studies, including our previous report, have provided evidence to show that it is directly or indirectly related [11–17, 19, 40].

In this study, no significant correlation was observed between bone density and the walking speed; however, BMI, one-leg standing and occlusal force were chosen as significant independent factors for bone density, other than age and sex. Vellas et al. reported that one-leg balance was a significant predictor of falling accidents [41], and it is known as an easy way to assess the physical performance of older adults. The lower limb muscle condition is directly related to daily activities such as walking, and therefore it has a strong correlation with physical activity in older adults [2]. It is common knowledge that a decreased muscle strength in the lower limbs is a risk factor for falling [42] and that a shorter one-leg standing time due to leg weakness and balance dysfunction means that the subject is more susceptible to falling [41]. A decreased walking speed is the most severe risk factor for events that are hazardous to older adults, such as falling [43, 44].

Our previous study showed that poor occlusal balance results in a shorter one-leg standing time, and occlusal force had a significant positive correlation with knee extension force [19]. Kimura et al. reported that dental treatment can extend the one-leg standing time [45]. Kimura et al. also reported that occlusal support when removable dentures are worn is related to Timed Up & Go Test and one-leg standing times [45]. Okada et al. [46] reported that a decrease in occlusal force was significantly associated with a decrease in walking speed, and some reports indicated a significant relationship between occlusal force and the physical performance [14, 15].

Based on the above evidence, we assume that good dental occlusion is positively associated with lower limb skeletal muscle strength, and that a stronger occlusal force leads to stronger skeletal muscle forces in the lower limbs and greater bone loading, resulting in a relatively higher bone density. Therefore, treatment for dentition and/or removable dentures which are able to apply proper occlusal force is assumed to be necessary to reduce the risk of falling.

An older age and/or female sex were identified as factors with an independent association with lower bone density, a weaker association between bone density and clinical characteristics, physical performance and the oral function was observed in females. Since it has been reported that the sex difference in tongue pressure [31] decreases at 60 years of age and older, and we found no sex differences in some of the variables in Table 1, we conducted a multiple regression analysis of the overall population, male subjects, and female subjects.

For females, it has been reported that they are more susceptible to falling than males and experience mild injuries related to falling [47, 48], in addition to it having been reported that the step variations of females render them more susceptible to falling compared to males [49].

We believe that the reason why age was an independent variable identified in females in this study is that the background factors for osteoporosis are more complex and confounded in females than in males. Risk factors for bone density loss include aging, lifestyle (ex. physical activity, alcohol intake, smoking, nutrition, lack of sun exposure), and heredity (ex. disease, a lean figure, family history), but the greatest risk factor is female sex [20, 22, 33, 50]. It is well known that a decrease in estrogen, a hormone produced by the ovarian follicles (only in females) has a significant effect on the bone metabolism, which is observed in bone density. The decline of estrogen in females begins in the postmenopausal period, that is, at the age of menopause, and bone density continues to decline in combination with aging [51]. This estrogen loss is not experienced by males who do not have ovaries, so the bone density in males only reflects the effects of risk factors other than estrogen decline. Ohta et al. reported that there is a marked sex difference in the pattern of bone density decline from approximately 45 years of age to 65 years of age, and there is no sex difference in the pattern of bone density decline after 65 years of age [50]. In other words, males experience a decrease in bone density due to the same mechanism as the decrease in skeletal muscle and occlusal force with aging. In females, the mechanism of age-related loss of skeletal muscle and occlusal force differs from that of males because the bone density shows a significant decrease after menopause due to aging. We suspect that the sex differences in this mechanism may have led to the results of this study. In fact, Tables 3 and 4, which contain the results of the simple comparison, showed that there were fewer variables in women than in men that showed a significant relationship with bone density. This study was a cross-sectional survey and only included individuals of 65 years of age and older; thus, this hypothesis regarding the cause of the sex difference is only speculative. An analysis of risk factors for bone density loss, which were not assessed in this study, may clarify the cause of this sex difference. At any rate, as osteoporosis progresses in female older adults following menopause, bone fractures caused by falling can progressively decrease their ADL or QOL, so we believe that taking measures to prevent falling in females is especially necessary.

One limitation associated with this study, this research is a cross-sectional study; therefore we would like to be able to clarify the causal relationships between aging changes in bone density and the oral function by future longitudinal analyses. The diagnosis of osteoporosis is actually diagnosed as osteoporosis regardless of the YAM value if there is a history of bone fragile vertebral body fracture / femoral fracture, but this study the fracture was not examined by X-ray and evaluated only by YAM. We did not assess factors that are closely related to bone density such as hormones and nutritional status in this study; however, we have obtained the data from the questionnaire [18]. The impact of the nutritional status is currently being analyzed and will be reported in the future.

## Conclusion

The bone density in the older adults showed a significant relationship not only with the clinical characteristics or physical performance but also with occlusal force. It may also be effective to confirm a good oral function in order to maintain healthy living for older adults.

## Data Availability

The materials described in the manuscript, including all relevant raw data, will be freely available to any scientist wishing to use them for non-commercial purposes, by contacting the corresponding author, without breaching patient confidentiality.

## Abbreviations

QOL: Quality of life
FESTA: Frail Elderly in the Sasayama-Tamba Area
BMI: Body mass index
SMI: Skeletal Muscle Mass Index
SOS: Speed of sound
YAM: a percentages of the young adult mean; walking speed, the normal walking speed; one-leg standing, standing on one leg with eyes open; remaining teeth, the number of remaining teeth; occlusal balance, the balance in occlusal force between the left and right sides
